# The Case for Assessing and Reporting on Facilitator Fidelity: Introducing the Fidelity of Implementation in Parenting Programs Guideline

**DOI:** 10.1007/s43477-023-00092-5

**Published:** 2023-09-09

**Authors:** Mackenzie Martin, Yulia Shenderovich, E. B. Caron, Justin D. Smith, Godfrey Siu, Susan M. Breitenstein

**Affiliations:** 1 Department of Social Policy and Intervention, University of Oxford, Oxford, UK; 2 Community-University Partnership for the Study of Children, Youth and Families, School of Public Health, University of Alberta, Edmonton, Canada; 3 Wolfson Centre for Young People’s Mental Health, Cardiff University, Cardiff, UK; 4 Centre for Development, Evaluation, Complexity and Implementation in Public Health Improvement (DECIPHER), School of Social Sciences, Cardiff University, Cardiff, UK; 5 Department of Psychology, University of Hartford, West Hartford, Connecticut USA; 6 Department of Population Health Sciences, Spencer Fox Eccles School of Medicine at the University of Utah, Salt Lake City, Utah USA; 7 Child Health and Development Centre, Makerere University, Kampala, Uganda; 8 College of Nursing, The Ohio State University, Columbus, Ohio USA

**Keywords:** Implementation, Fidelity, Parenting, Reporting, Scale, Commentary

## Abstract

The sizeable body of evidence indicating that parenting programs have a positive impact on children and families highlights the potential public health benefits of their implementation on a large scale. Despite evidence and global attention, beyond the highly controlled delivery of parenting programs via randomized trials, little is known about program effectiveness or how to explain the poorer results commonly observed when implemented in community settings. Researchers, practitioners, and policymakers must work together to identify what is needed to spur adoption and sustainment of evidence-based parenting programs in real-world service systems and how to enhance program effectiveness when delivered via these systems. Collecting, analyzing, and using facilitator fidelity data is an important frontier through which researchers and practitioners can contribute. In this commentary, we outline the value of assessing facilitator fidelity and utilizing the data generated from these assessments; describe gaps in research, knowledge, and practice; and recommend directions for research and practice. In making recommendations, we describe a collaborative process to develop a preliminary guideline—the Fidelity of Implementation in Parenting Programs Guideline or FIPP—to use when reporting on facilitator fidelity. Readers are invited to complete an online survey to provide comments and feedback on the first draft of the guideline.

**Supplementary Information:**

The online version contains supplementary material available at 10.1007/s43477-023-00092-5.

## Introduction

Parenting programs empower parents/caregivers (“parents”) to acquire the knowledge and/or skills to support them to improve the health and well-being of their children (Barlow & Coren, 2018). There are hundreds of randomized trials and numerous meta-analyses indicating that parenting programs are effective in improving child and family outcomes, including reducing child behavior problems and violence against children and improving positive parenting and child mental health (Barlow & Coren, 2018; Barlow et al., 2006; Chen & Chan, 2016; Furlong, 2013). For instance, a meta-analysis of 37 trials of parenting programs aiming to reduce child maltreatment had an effect size of 0.30 (Chen & Chan, 2016) and a meta-analysis of 45 trials of parenting programs aiming to treat child disruptive behaviors had an effect size of 0.69 (Leijten et al., 2019). Several ‘reviews of reviews’ have drawn similar conclusions (Barlow & Coren, 2018; Coore Desai et al., 2017; Mikton & Butchart, 2009; Sandler et al., 2011, 2015). Finally, there is meta-analytic evidence to suggest that parenting programs are effective when transported to new delivery systems and cultural contexts (Gardner et al., 2016; Smith et al., 2020).

The sizeable body of evidence indicating that parenting programs have a positive impact on children and families highlights the importance of their implementation on a large scale. Several influential organizations recommend that parenting programs be delivered at scale to empower parents in enhancing child development, strengthening families, and promoting the safety and well-being of children (Institute of Medicine & National Research Council, 2014; National Academies of Sciences, 2022; World Health Organization, 2022). For instance, the World Health Organization recommends parenting programs as one of seven strategies for reducing violence against children (WHO, 2016). If implemented broadly, parenting programs could be a key means through which to attain several of the 2030 Sustainable Development Goals (e.g., Goals 3 and 16) and actualize the Convention on the Rights of the Child (e.g., Articles 5 and 19) (Eisner et al., 2016).

### Science to Service

Despite the evidence and global attention, there is a gap between what is known about the effectiveness of parenting programs when delivered via randomized trials and the extent to which they are delivered in practice via community settings. This “science to service gap” (Fixsen et al., 2009) is commonly acknowledged in the literature, particularly at scale (Gottfredson et al., 2015) and in low- and middle-income contexts where violence against children is prevalent (Hillis et al., 2016; Knerr et al., 2013; Shenderovich, 2021; Stoltenborgh et al., 2013). Among the few studies that examine scale-up, there has been mixed evidence of program effectiveness (e.g., Gray et al., 2018; Marryat et al., 2017; Shapiro et al., 2010). Evidence on scaling from similar interventions, such as on early childhood development programs, suggest effects at scale may be smaller including due to lower quality of delivery and problems with staff retention (Araujo et al., 2021)—a phenomenon described as the “scale-up penalty” (Institute of Medicine & National Research Council, 2014). In the parenting program literature, an evaluation of the Triple P program in Scotland found that the intervention did not impact child mental health when delivered at the population level (Marryat et al., 2017), whereas a study of the Incredible Years and Triple P programs in England found that the interventions had a similarly positive impact on child behavior, parenting behavior, and parental mental health when delivered in both research and community settings (Gray et al., 2018). Another example of successful delivery at scale is the Parent Management Training-Oregon Model program, which has demonstrated the ability to be transported to new cultural contexts (Ogden & Hagen, 2008) and delivered at scale in Norway with implementation fidelity supports and monitoring (Askeland et al., 2019). Overall, beyond the highly controlled delivery of parenting programs via randomized trials, little is known about program effectiveness or how to explain the poorer results commonly observed when implemented in community settings. Although we currently do not know how to account for these gaps, parenting programs have tremendous potential to be a positive force for children and families. As a result, researchers, practitioners, and policymakers must work together to identify what is needed to spur adoption and sustainment of parenting programs in real-world service systems and how to enhance program effectiveness when delivered via these systems (Smith et al., 2020).

### Bridging the Gap

One of several avenues to bridge the gap in our knowledge is the use of implementation fidelity monitoring (Forgatch & DeGarmo, 2011). Implementation fidelity refers to the extent to which an intervention is implemented as intended (Bumbarger & Perkins, 2008; Dane & Schneider, 1998; Dusenbury et al., 2003). There are four commonly acknowledged components of implementation fidelity—adherence, quality of delivery (or competence), dosage, and participant responsiveness (Dane & Schneider, 1998; Dusenbury et al., 2003; Mihalic, 2004; Proctor et al., 2011). Herein, we focus on two aspects of implementation fidelity directly related to the delivery of parenting programs by facilitators (i.e., delivery agents, purveyors, therapists, group leaders)—adherence (the strictness with which a facilitator implements program components as intended) and competence (the skill and style with which a facilitator delivers program components) (Dane & Schneider, 1998; Dusenbury et al., 2003; Fixsen et al., 2005). As Berkel et al. (2019) highlight, competence and adherence, also referred to as ‘competent adherence’, ‘facilitator fidelity’, and ‘facilitator delivery’ (Breitenstein et al., 2010a, 2010b; Forgatch et al., 2005), tend to suffer when programs are delivered via routine service delivery due in part to a lack of a formal implementation support mechanisms.

### Facilitator Fidelity

The competent adherence with which facilitators implement parenting programs is a particularly important aspect of implementation fidelity to examine as facilitators are the vehicle through which participants receive an intervention (Petersilia, 1990). Many studies indicate that higher quality delivery of parenting programs by facilitators is associated with improved parent and child outcomes (e.g., Chiapa et al., 2015; Eames et al., 2008; Forgatch & DeGarmo, 2011; Forgatch et al., 2005; Martin et al., 2023; Scott et al., 2008) and is associated with critical process variables for prevention programs, such as motivation and engagement (Berkel et al., 2021; Smith et al., 2013). Despite a large body of evidence on parenting programs, relatively little is known about the quality with which facilitators implement parenting programs in practice and when delivered at scale (Smith et al., 2019). This gap in knowledge is in part due to few studies on the subject and the lack of consistency of reporting among studies that examine facilitator delivery (Martin et al., 2021, 2023). Closing the facilitator fidelity knowledge gap would go a long way toward generating understanding and concerning the differences in effectiveness found between parenting programs delivered in randomized trials and parenting programs delivered in real-world settings, particularly at scale.

To support the argument that parenting program research and practice should devote greater attention to assessing facilitator fidelity, we outline the value of assessing facilitator fidelity and using the data generated from these assessments; describe gaps in research, knowledge, and practice related to assessing facilitator fidelity; and recommend directions for research and practice. In making these recommendations, we describe a collaborative process to develop a preliminary guideline for parenting program researchers—the Fidelity of Implementation in Parenting Programs Guideline—to use when reporting on facilitator fidelity. As part of this process, readers are invited to complete an online survey to provide comments and feedback on the first draft of the guideline.

## Assessing and Reporting Facilitator Fidelity

The systematic assessment and comprehensive reporting of facilitator fidelity would be of substantial value to the parenting program field by contributing to knowledge in five key areas related to research on both the efficacy of such programs and their implementation in practice. Data on facilitator fidelity provides critical information about:

1. the extent to which program theory is implemented in practice (Breitenstein et al., 2010a, 2010b). Even though a program may have a strong theoretical foundation or is efficacious in randomized trials, it does not necessarily mean that it will be delivered as planned in practice or that it will be used as expected by the intended stakeholders (Petersilia, 1990). Thus, fidelity data support a determination of the magnitude of Type III error (Carroll et al., 2007; Dobson & Cook, 1980).
2. the potential mechanisms through which an intervention affects its outcomes (Astbury & Leeuw, 2010; Berkel et al., 2019; Fixsen et al., 2005; Scriven, 1999). Uncovering such mechanisms may illuminate what program components are and are not contributing to the achievement of the outcomes found so that participant outcomes can be maximized by delivering essential components and shedding unessential components (Van Ryzin et al., 2016).
3. how interventions and implementation can be improved (Breitenstein et al., 2010a, 2010b). For instance, fidelity data can help establish what components facilitators struggle to deliver, which can inform ongoing and future training and coaching processes.
4. whether implementation fidelity is associated with participant outcomes. Higher program implementation quality—such as participant attendance, participant engagement in sessions, and delivery by facilitators—is theorized and commonly found to be associated with enhanced participant outcomes in the broader behavioral intervention literature (Carroll et al., 2007; Durlak & DuPre, 2008). Similar findings have emerged in the parenting program literature (Leitão et al., 2020; Martin et al., 2023).
5. what types of supervision models and related implementation supports are needed to dependably deliver a high-quality intervention in various contexts during intervention dissemination and scale-up (Glasgow et al., 2003). This is especially the case as intervention effectiveness is often weakened at scale due to poor implementation or drift, which can occur once interventions become widely used (Bond et al., 2000; Botvin, 2004). ‘Drift’ is distinct from ‘adaptation’; the latter occurs when planned or unplanned changes are made to a program or its delivery to suit the context (Campbell et al., 2020). While adaptations are often conceptualized as detracting from an intervention, there is a growing recognition that varying degrees of adaptation may be necessary to maintain functional fidelity, such as in a new context (Moore et al., 2021).

The value that facilitator fidelity provides points toward collecting this data via facilitator assessments as a next frontier through which researchers and practitioners can advance parenting intervention science. However, there are several gaps and challenges associated with assessing facilitator fidelity in both research and practice that should be considered and addressed.

## Gaps and Challenges

Related to research, there are gaps in knowledge as well as in reporting. First, there is scant literature on the reliability and validity of facilitator fidelity measures—as is evidenced by few studies examining the psychometric properties of measures used to assess facilitator fidelity (Martin et al., 2021). Although a lack of psychometric evidence may only seem like a concern to researchers, it is also problematic for practitioners as they too use assessment results to inform decision-making and the allocation of often scarce resources for training and supervision. Without reliable and valid measures, decisions using fidelity data cannot be made with a great deal of confidence. In sum, reliable and valid measures are essential to their use (Ruud et al., 2020).

Second, rigorous measures designed and used in the context of efficacy trials are often impractical for use in community settings and at scale. For facilitator fidelity assessments to be used in practice, considerable trade-offs are necessary to balance research rigor and real-world practicality. Although observational measures are considered most rigorous, these methods pose significant practical challenges especially at scale (Eames et al., 2008). In addition to being time and resource intensive, observational assessments may be impacted by reactivity bias (Girard & Cohn, 2016). Although non-observational methods do not use as many human resources (i.e., time and money), they may miss important aspects of delivery (e.g., body language, participation reactions, participant engagement) and their reliability may be limited by social desirability (Stone et al., 1999). The tension between rigor and practicality appears most apparent as programs transition from delivery via efficacy and effectiveness trials to implementation via routine service delivery and at scale.

Third, few studies report on facilitator fidelity in detail. In research papers, details on the reporting of how assessments are conducted is particularly lacking, such as information about the characteristics of the facilitators delivering programs; the educational background and measure-specific training for those who conduct facilitator assessments (assessors or coders); and the amount of time and money necessary to complete fidelity assessments (Martin et al., 2021). These details regarding assessments are most relevant beyond efficacy trials in the context of real-world implementation when resource limitations are even more poignant. Further, describing the real-world logistics (e.g., audio/video recording, observation) of conducting fidelity assessments is important as compiling a body of literature may allow for the advancement of knowledge on how to make fidelity assessments easier and less costly, particularly at scale (Lewis et al., 2021). Similarly, few studies examine or report on the association between facilitator fidelity and outcomes (Martin et al., 2021, 2023). Among the studies that do report on facilitator fidelity in general, the reporting of key information about facilitator fidelity is inconsistent. For instance, many studies do not provide details regarding facilitator sample sizes and average level of delivery fidelity achieved (Martin et al., 2021)—information necessary for meta-analyzing the literature, assessing the strength of study findings, and determining the extent to which programs are delivered in practice. Additionally, without evidence that facilitator fidelity predicts program outcomes, the value proposition of investing resources in the measurement and use of fidelity data is undetermined.

As it relates to practice, while insufficient attention is paid to facilitator delivery in research, there is even less focus on fidelity monitoring once interventions are implemented in community settings. This is understandable as fidelity monitoring is typically an extremely time and resource intensive process with current methods (Anis et al., 2021). Fidelity monitoring is complicated as it involves many steps, including developing an appropriate measure, testing the measure, training individuals to use the measure (assessors), having assessors use the measure in practice, providing ongoing supervision to facilitators being assessed using the measure, tabulating assessment data, and then using the assessment data collected to inform program improvements (e.g., Sanders et al., 2020). These steps not only take time and energy but are costly thereby requiring substantial budgets. A particular challenge with facilitator assessments is that measures are often quite detailed and lengthy. However, little is known regarding how best to develop and implement fidelity assessments. For instance, there is insufficient understanding of how measures should capture the tension between fidelity and adaptation, which is important as both planned and unplanned adaptations are typically made in practice and at scale (Axford et al., 2017; Kemp, 2016; Lize et al., 2014).

Thus, there are many challenges and gaps in research and practice related to assessing facilitator fidelity that need to be addressed. Although some of the challenges in assessing facilitator fidelity overlap between controlled evaluations and implementation in practice, some aspects are unique to specific stages of the research to practice pipeline.

## Recommendation for Future Research and Practice

We recommend several approaches to advance future research and practice in parenting program implementation overall and at three points on the research translation pipeline.

### Overall

For parenting programs at a community level, we recommend creating a ‘fidelity culture’ among researchers, implementing organizations, assessors, and facilitators. Such a culture appears essential as fidelity monitoring requires substantial organizational commitment (Axford et al., 2017). Various actions could be taken to support a fidelity culture including designing measures with practicality in mind (Lewis et al., 2021). Relatedly, facilitating such a supportive environment is important as there is understandably some resistance and anxiety among many implementing staff regarding being evaluated. This resistance and anxiety stems from the potentially critical and punitive nature of assessments rather than a lack of agreement on the value of assessments. As a result, implementing staff conducting evaluations and being evaluated need to be assured and feel that the process is supportive and collaborative.

### Pre-intervention

Before parenting programs are delivered, we recommend

1. Documenting the program theory of change (logic model) and highlighting the core components and mechanisms of the program that should be followed to retain fidelity (Moore et al., 2021).
2. Designing fidelity measures and assessment processes that will provide valuable information to various stakeholders, not just researchers. To do so, measures might be designed by engaging stakeholders in a content validity exercise (e.g., Martin et al., 2022).
3. Establishing procedures to collect fidelity assessment data as well as relevant information associated with the fidelity assessment process (e.g., documenting time required per assessor and per trainer to complete assessor training).
4. Preparing to collect data that can be used to establish the reliability and validity of the measure designed or chosen to assess facilitator fidelity (e.g., determining intra- and inter-rater reliability during assessor training).
5. Reporting how facilitator fidelity assessment data will be collected and sharing such information via study protocols.

### Efficacy and Effectiveness Trials

When parenting programs are tested via efficacy and effectiveness trials, we recommend

1. Reporting the results of fidelity assessments to advance the literature on the extent and quality with which parenting programs are delivered under ideal circumstances.
2. Conducting and reporting on analyses of measure reliability and validity (Stirman, 2020). Gathering and synthesizing psychometric evidence is critical to ensuring measures and assessments using these measures are of good quality so that subsequent critical decision-making about both future trials and program delivery in practice is based on solid data.
3. Conducting and reporting on analyses of the relationship between facilitator fidelity and program outcomes. A better understanding of this relationship could lend insight into the mechanisms through which parenting programs work, which can then inform decision-making about how to best dedicate scarce resources to maximize participant outcomes.
4. Investigating what constitutes a sufficient facilitator delivery monitoring process to inform how fidelity assessments can be made easier to conduct in practice. Further recommendations to this end include:
  i. testing different assessment procedures to determine whether simplified methodologies are sufficient (e.g., Suhrheinrich et al. (2020) compared a three- or five-point Likert scale in an attempt to create a measure that was both useable and rigorous);
  ii. establishing whether simpler data collection processes—particularly self-report tools—are reliable (e.g., Tiwari et al. (2021) compared audio assessments with video assessments and found 72% agreement; Breitenstein et al., (2010a, 2010b) compared in-person observations with self-reports and found 85% agreement);
  iii. examining whether assessments conducted at one timepoint are representative of overall delivery (e.g., Caron et al. (2018) examined the impact of facilitators’ average delivery across cases, as well as case-specific delivery; Shenderovich et al. (2019) examined how facilitator delivery fluctuated over 14 sessions);
  iv. studying how facilitator delivery varies over time, particularly drift over longer periods of time and over many years, such as has been examined in the Parent Management Training-Oregon Model (Forgatch & DeGarmo, 2011) and Family Check-Up® programs (Chiapa et al., 2015) and
  v. exploring and evaluating novel methods of assessing facilitator delivery, such as qualitative or automated methods (e.g., Berkel, Smith, et al. [under review] explore the potential of a machine learning approach based on natural language processing to capture facilitator fidelity).
5. In collaboration with relevant stakeholders, establishing processes for translating information on fidelity assessments into practice by delineating the explicit feedback loops through which knowledge of the strengths and weaknesses of facilitator delivery can improve practice via program manuals, facilitator training, and ongoing supervision (Fixsen et al., 2005).

### Dissemination and Implementation in Real-World Delivery Systems

When parenting programs are disseminated and scaled within real-world delivery systems, we recommend:

1. Implementing the knowledge gained on fidelity monitoring from efficacy and effectiveness trials (e.g., using the results of fidelity assessments to modify facilitator training materials).
2. Establishing tools and processes to monitor facilitator fidelity and then using the processes and procedures to monitor facilitator fidelity throughout delivery.
3. Evaluating whether fidelity continues to be associated with program outcomes in community settings.
4. Investigating whether fidelity assessment tools and processes are practical to implement, such as using the Psychometric and Pragmatic Evidence Rating Scale (PAPERS), which specifies criteria to evaluate implementation measures (Lewis et al., 2021). Information about measure practicality may also be captured via qualitative methods to ascertain facilitator and assessor experiences of fidelity assessments.
5. Collaborating with stakeholders to analyze the facilitator fidelity data collection procedure and then apply the findings to inform future evaluations and modifications to fidelity monitoring processes.

## Reporting Guidelines: The FIPP Guideline

The implementation of many of the above recommendations would be supported by rigorous reporting of fidelity monitoring at all stages in the research translation pipeline. As a result, the authors are in the process of creating a reporting guideline on facilitator fidelity assessment and reporting practices, the FIPP guideline, for use by researchers and practitioners studying and implementing parenting programs aiming to strengthen parenting practices and reduce child behavior problems and violence against children. We draw on the recommendations published by Moher et al. (2010) on the development of reporting guidelines. In sum, a multi-part online Delphi exercise is being employed. First, relevant literature has been reviewed to develop a preliminary draft of the FIPP (see Table 1). The draft reflects the types of information that were articulated as important in a range of articles published in the parenting and broader behavioral intervention literature (Bellg et al., 2004; Breitenstein et al., 2010a, 2010b; Breitenstein et al., 2010a, 2010b; Martin et al., 2021, 2023). The guideline was also created based on the expertise and experience of the co-authors, who are involved in fidelity assessment in the research and practice of parenting programs around the world, including Parenting for Lifelong Health, the Chicago Parent Program, Family Check-Up®/Family Check-Up® 4 Health, Parenting for Respectability, and Attachment and Biobehavioral Catch-Up. Based on the literature and the experience of the authors, the draft guideline includes six sections recommending that those reporting on facilitator fidelity provide details on intervention characteristics, facilitator characteristics, assessor characteristics, measure characteristics, fidelity results, and potential biases. Second, the draft guideline will be shared with parenting experts and practitioners to gather their input via an online survey, including via the publication of this article. The authors invite readers to provide input on the draft guideline by completing an anonymous online survey. Third, the draft guideline included in this commentary will be revised based on the results of the online survey. Fourth, a consensus meeting will be held with invited researchers and practitioners to discuss, edit, and vote on each item included in the guideline. Fifth, a reporting guideline will be created and published for researchers and practitioners in the parenting field to consider, use, and further revise. As the guideline does not include items on program adaptations, it is envisaged that the guideline developed herein could be used in conjunction with frameworks and reporting guidelines specific to adaptations (e.g., FRAME-IS) (Miller et al., 2021).

**Table 1:** Preliminary draft of the fidelity of implementation in parenting programs guideline

Category	Reporting Items
Intervention characteristics	1. Identify the program title/brand 2. Identify the program aim(s) 3. Identify the theorized core components of the intervention 4. Explain the program mode of delivery covered by the fidelity assessment (e.g., home visits, group sessions) 5. Identify the number of sessions 6. Describe the target population for the intervention 7. Describe facilitator training, certification, and supervision for the intervention 8. Specify the country of program delivery
Intervention Facilitator Characteristics	9. Document the demographic characteristics of the facilitator sample 10. Describe the relevant experience and education of facilitators 11. Specify the sample size of facilitators being assessed 12. Specify the average number of observations per facilitator with the standard deviation and range
Fidelity assessor characteristics	13. Identify who conducted the assessments 14. Identify any assessor educational background requirements 15. Describe assessor training requirements
Fidelity measure characteristics	16. Identify the name of the measure 17. Describe the development process used to create the measure 18. Describe how the program theory has informed the fidelity measure 19. Specify the construct(s) of implementation fidelity measured (e.g., competence, adherence, both) 20. Specify the mode through which assessments are collected (e.g., video, audio, live, self-report) 21. Specify the type of response option used (e.g., Likert scale, dichotomous, notes, counts) 22. Explain the session sampling technique (e.g., random selection, self-selection by facilitator, convenience), and if session segments (specify length) or full sessions were assessed 23. Describe the timeline of fidelity assessments (e.g., throughout data collection, after data collection has finished) 24. Describe processes used to provide facilitators with feedback (e.g., individual-level, group-level) 25. Describe notable changes in implementation made as a result of ongoing fidelity data results 26. Comment on any studies which present data on the reliability and validity of the measure 27. If possible, present evidence on the reliability (i.e., intra-rater reliability, inter-rater reliability, internal consistency, test–retest reliability/dependability) and validity (i.e., content validity, construct validity) of the measure
Fidelity results	28. If possible given the type of measure used, by type of fidelity measured (e.g., adherence, competence, both), provide a breakdown of the average results with standard deviations in percentages or in a format that can easily be converted to a percentage 29. If possible given the type of measure used, by type of fidelity measured, report the range of results in percentages or in a format that can easily be converted to a percentage 30. If possible, broken down by type of fidelity measured, report unadjusted associations with parent and/or child outcomes (i.e., bivariate correlations and unstandardized regressions)
Bias	31. Report study pre-registration details 32. Identify factors that may increase or decrease the objectivity in assessments (e.g., describe the extent to which assessors are able to make independent and objective assessments; supervisory or other relationships between the assessors and facilitators) 33. Describe the degree to which facilitators could be reactive to being assessed 34. Describe the extent to which self-desirability or job performance evaluations could influence assessments

## Conclusion

Evidence indicates that parenting programs have great potential to improve the health and well-being of children and families around the world. However, further implementation research is needed regarding how parenting programs might be effectively delivered as part of routine service delivery and at scale. Monitoring, analyzing, and reporting facilitator fidelity data will be a key driver of the parenting program community’s continuing and further success to ensure the efficient use of resources to achieve maximal public health benefit from parenting programs. This commentary describes the advantages of assessing and using data on facilitator delivery, outlines gaps in current knowledge, recommends future avenues for research and practice with parenting program fidelity, and sets out the process being used to create the FIPP—a reporting guideline for the parenting program community to use to ensure consistency in reporting facilitator assessment data.

Readers are encouraged to contribute to the FIPP reporting guideline by offering their feedback here: https://osu.az1.qualtrics.com/jfe/form/SV_29Nftj6wkrnVbwi (see Supplementary File 2 for QR code).

### Supplementary Information

Below is the link to the electronic supplementary material.

Supplementary file1 (DOCX 34 kb)

Supplementary file2 (PNG 1 kb)
