# Supplementary material for: The Case for Assessing and Reporting on Facilitator Fidelity: Introducing the Fidelity of Implementation in Parenting Programs Guideline
Source: Glob Implement Res Appl. 2023 Sep 9;4(1):1–10. doi: 10.1007/s43477-023-00092-5 (PMC10873439; doi:10.1007/s43477-023-00092-5)
Supplement: Supplementary file 1 — Supplementary file1 (DOCX 34 kb) [file 43477_2023_92_MOESM1_ESM.docx]

**FIDELITY OF IMPLEMENTATION IN PARENTING PROGRAMS SURVEY**

*What is this survey about?*

The purpose of this survey is to assess your views on a draft list of items to include in a Fidelity of Implementation in Parenting Programs statement. We hope this statement can be used by researchers and practitioners implementing and evaluating parenting programs.

In this survey, you will be given a list of potential items that could be included in the statement. Once you have completed the survey, you can indicate whether you would like to participate in a consensus meeting later this year to refine and finalize the reporting guideline.

*How do I complete this survey?*

This survey has 30 questions and should take approximately 15-20 minutes to complete. Please rate each proposed item on a scale of 1 to 3 wherein 1 indicates that an item is not important and thus should not be included in the final scale; 2 indicates that an item may or may not be important and you are not sure whether it should be included; and 3 indicates that an item is very important and thus should be included.

In answering each question, please reflect on the content included in the question rather than the exact wording of the question. The wording of each item will be refined and finalized at the consensus meeting. Each item will also include a text box where you can write your suggested revisions to the proposed items. Items which predominantly receive a “2” will be further discussed during the consensus meeting. At the end of the survey, you will have the opportunity to propose additional items to be included in the guideline.

**SURVEY:**

**CATEGORY 1 – Intervention Characteristics**

1. Identify the program title/brand
   - 1 – not important
   - 2 – not sure
   - 3 – very important

-comment box-

1. Identify the program aim(s)
   - 1 – not important
   - 2 – not sure
   - 3 – very important

-comment box-

1. Identify the theorized core components of the intervention
   - 1 – not important
   - 2 – not sure
   - 3 – very important

-comment box-

1. Explain the program mode of delivery covered by the fidelity assessment (e.g., home visits, group sessions)
   - 1 – not important
   - 2 – not sure
   - 3 – very important

-comment box-

1. Identify the number of sessions
   - 1 – not important
   - 2 – not sure
   - 3 – very important

-comment box-

1. Describe the target population for the intervention
   - 1 – not important
   - 2 – not sure
   - 3 – very important

-comment box-

1. Describe facilitator training, certification, and supervision for the intervention
   - 1 – not important
   - 2 – not sure
   - 3 – very important

-comment box-

1. Specify the country of program delivery
   - 1 – not important
   - 2 – not sure
   - 3 – very important

-comment box-

**CATEGORY 2 – Intervention Facilitator Characteristics**

1. Document the demographic characteristics of the facilitator sample
   1. 1 – not important
   2. 2 – not sure
   3. 3 – very important

-comment box-

1. Describe the relevant experience and education of facilitators
   - 1 – not important
   - 2 – not sure
   - 3 – very important

-comment box-

1. Specify the sample size of facilitators being assessed
   - 1 – not important
   - 2 – not sure
   - 3 – very important

-comment box-

1. Specify the average number of observations per facilitator with the standard deviation and range
   - 1 – not important
   - 2 – not sure
   - 3 – very important

-comment box-

**CATEGORY 3 – Fidelity Assessors Characteristics**

1. Identify who conducted the assessments
   - 1 – not important
   - 2 – not sure
   - 3 – very important

-comment box-

1. Identify any assessor educational background requirements
   - 1 – not important
   - 2 – not sure
   - 3 – very important

-comment box-

1. Describe assessor training requirements
   - 1 – not important
   - 2 – not sure
   - 3 – very important

-comment box-

**CATEGORY 4 – Fidelity Measure Characteristics**

1. Identify the name of the measure
   - 1 – not important
   - 2 – not sure
   - 3 – very important

-comment box-

1. Describe the development process used to create the measure
   - 1 – not important
   - 2 – not sure
   - 3 – very important

-comment box-

1. Describe how the program theory has informed the fidelity measure
   - 1 – not important
   - 2 – not sure
   - 3 – very important

-comment box-

1. Specify the construct(s) of implementation fidelity measured (e.g., competence, adherence, both)
   - 1 – not important
   - 2 – not sure
   - 3 – very important

-comment box-

1. Specify the mode through which assessments are collected (e.g., video, audio, live, self-report)
   - 1 – not important
   - 2 – not sure
   - 3 – very important

-comment box-

1. Specify the type of response option used (e.g., Likert scale, dichotomous, notes, counts)
   - 1 – not important
   - 2 – not sure
   - 3 – very important

-comment box-

1. Explain the session sampling technique (e.g., random selection, self-selection by facilitator, convenience), and if session segments (specify length) or full sessions were assessed
   - 1 – not important
   - 2 – not sure
   - 3 – very important

-comment box-

1. Describe the timeline of fidelity assessments (e.g., throughout data collection, after data collection has finished)
   - 1 – not important
   - 2 – not sure
   - 3 – very important

-comment box-

1. Describe processes used to provide facilitators with feedback (e.g., individual-level, group-level)
   - 1 – not important
   - 2 – not sure
   - 3 – very important

-comment box-

1. Describe notable changes in implementation made as a result of ongoing fidelity data results
   - 1 – not important
   - 2 – not sure
   - 3 – very important

-comment box-

1. Comment on any studies which present data on the reliability and validity of the measure
   - 1 – not important
   - 2 – not sure
   - 3 – very important

-comment box-

1. If possible, present evidence on the reliability (i.e., intra-rater reliability, inter-rater reliability, internal consistency, test re-test reliability/dependability) and validity (i.e., content validity, construct validity) of the measure
   - 1 – not important
   - 2 – not sure
   - 3 – very important

-comment box-

**CATEGORY 5 – Results (Level of fidelity reported)**

1. If possible given the type of measure used, by type of fidelity measured (e.g., adherence, competence, both), provide a breakdown of the average results with standard deviations in percentages or in a format that can easily be converted to a percentage
   - 1 – not important
   - 2 – not sure
   - 3 – very important

-comment box-

1. If possible given the type of measure used, by type of fidelity measured, report the range of results in percentages or in a format that can easily be converted to a percentage
   - 1 – not important
   - 2 – not sure
   - 3 – very important

-comment box-

1. If possible, broken down by type of fidelity measured, report unadjusted associations with parent and/or child outcomes (i.e., bivariate correlations and unstandardized regressions)
   - 1 – not important
   - 2 – not sure
   - 3 – very important

-comment box-

**CATEGORY 6 – Bias**

1. Report study pre-registration details
   - 1 – not important
   - 2 – not sure
   - 3 – very important

-comment box-

1. Identify factors that may increase or decrease the objectivity in assessments (e.g., describe the extent to which assessors are able to make independent and objective assessments; supervisory or other relationships between the assessors and facilitators)
   - 1 – not important
   - 2 – not sure
   - 3 – very important

-comment box-

1. Describe the degree to which facilitators could be reactive to being assessed
   - 1 – not important
   - 2 – not sure
   - 3 – very important

-comment box-

1. Describe the extent to which self-desirability or job performance evaluations could influence assessments
   - 1 – not important
   - 2 – not sure
   - 3 – very important

-comment box-

**MISSING QUESTIONS**

If you believe any questions are missing from the above, please write them in the box provided below.

-comment box-

**RESPONDENT-SPECIFIC QUESTIONS**

Which category best describes your expertise as it relates to parenting programs? (please select all that apply)

- - Researcher/scientist
  - Staff member at an organization coordinating the delivery of one or more parenting programs
  - Parenting program facilitator
  - Parenting program coach/supervisor/consultant
  - Assessor of facilitator delivery
  - Developer of a parenting program
  - I do not have experience related to parenting programs
  - Other (please specify):

-comment box-

Would you like to be involved in a consensus meeting to discuss and refine this reporting guideline? This meeting is expected to take up approximately 1.5-2 hours of your time.

- - Yes (please provide in the comment box below)
  - No

-comment box-
