# Supplementary figures and images for: The Case for Assessing and Reporting on Facilitator Fidelity: Introducing the Fidelity of Implementation in Parenting Programs Guideline
Source: Glob Implement Res Appl. 2023 Sep 9;4(1):1–10. doi: 10.1007/s43477-023-00092-5 (PMC10873439; doi:10.1007/s43477-023-00092-5)

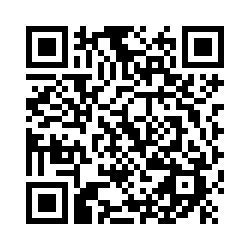

Supplement: Supplementary file 2 — Supplementary file2 (PNG 1 kb) [file 43477_2023_92_MOESM2_ESM.png]
